# Identification and antimicrobial resistance patterns of bacterial enteropathogens from children aged 0–59 months at the University Teaching Hospital, Lusaka, Zambia: a prospective cross sectional study

**DOI:** 10.1186/s12879-017-2232-0

**Published:** 2017-02-02

**Authors:** Harriet Chiyangi, John B. Muma, Sydney Malama, Joel Manyahi, Ahmed Abade, Geoffrey Kwenda, Mecky I. Matee

**Affiliations:** 1 Department of Epidemiology and Biostatistics, School of Public Health and Social Sciences, Muhimbili University of Health and Allied Science, Dar es Salaam, Tanzania; 2 Tanzania Field Epidemiology and Laboratory Management Program, Ministry of Health, Dar es Salaam, Tanzania; 3 Department of Disease Control, School of Veterinary, University of Zambia, Lusaka, Zambia; 4 Health Promotions Research Program, Institute of Economic and Social Research, University of Zambia, Lusaka, Zambia; 5 Department of Biomedical Sciences, School of Health Sciences, University of Zambia, Lusaka, Zambia; 6 Department of Microbiology and Immunology, School of Medicine, Muhimbili University of Health and Allied Science, Dar es Salaam, Tanzania

**Keywords:** Bacteria, Diarrhea, Children, Antimicrobial, Zambia

## Abstract

**Background:**

Bacterial diarrhoeal disease is among the most common causes of mortality and morbidity in children 0–59 months at the University Teaching Hospital in Lusaka, Zambia. However, most cases are treated empirically without the knowledge of aetiological agents or antimicrobial susceptibility patterns. The aim of this study was, therefore, to identify bacterial causes of diarrhoea and determine their antimicrobial susceptibility patterns in stool specimens obtained from the children at the hospital.

**Methods:**

This hospital-based cross-sectional study involved children aged 0–59 months presenting with diarrhoea at paediatrics wards at the University Teaching Hospital in Lusaka, Zambia, from January to May 2016. Stool samples were cultured on standard media for enteropathogenic bacteria, and identified further by biochemical tests. Multiplex polymerase chain reaction was used for characterization of diarrhoeagenic *Escherichia coli* strains. Antimicrobial susceptibility testing was performed on antibiotics that are commonly prescribed at the hospital using the Kirby-Bauer disc diffusion method, which was performed using the Clinical Laboratory Standards International guidelines.

**Results:**

Of the 271 stool samples analysed *Vibrio cholerae* 01 subtype and Ogawa serotype was the most commonly detected pathogen (40.8%), followed by *Salmonella* species (25.5%), diarrhoeagenic *Escherichia coli* (18%), *Shigella* species (14.4%) and *Campylobacter* species (3.5%). The majority of the bacterial pathogens were resistant to two or more drugs tested, with ampicillin and co-trimoxazole being the most ineffective drugs. All diarrhoeagenic *Escherichia coli* isolates were extended spectrum β-lactamase producers.

**Conclusion:**

Five different groups of bacterial pathogens were isolated from the stool specimens, and the majority of these organisms were multidrug resistant. These data calls for urgent revision of the current empiric treatment of diarrhoea in children using ampicillin and co-trimoxazole, and emphasizes the need for continuous antimicrobial surveillance as well as the implementation of prevention programmes for childhood diarrhoea.

## Background

Infectious diarrhoea is a significant cause of illness and death among children under 5 years of age in low-resource countries. It accounts for 9% of all deaths globally in this age group, and ranks only second to pneumonia [1]. The majority of these cases are associated with the first two years of life, with peak ages being between 6 and 11 months [1, 2]. Although mortality associated with diarrhoea has been decreasing since 2000, mainly due to the implementation of effective control programmes and improved socioeconomic status, it still remains an important reason for hospital admissions and deaths among the children [3]. South-East Asia and sub-Saharan Africa bear the highest burden of the disease [1, 4].

Interventions that target the main causes of diarrhoea should focus on the most susceptible children, and this should further accelerate decline of diarrhoeal cases. Guiding these efforts requires identification of aetiological agents and understanding the risk factors associated with diarrhoea. Most cases of diarrhoea are associated with consumption of contaminated water and food, and poor sanitation, which create an ideal environment for diarrhoeal pathogens to be easily transmitted [2, 5]. Several pathogens have been implicated as important causes of diarrhoea, and these include a variety of bacteria, parasites and viruses [4, 6, 7].

Although the most effective treatment for acute diarrhoea is fluid and electrolyte replacement, antibacterial agents are often indicated in dysentery, typhoid fever and severe cholera [3, 8]. However, in recent years there has been growing concern of antimicrobial resistance in bacterial pathogens associated with diarrhoea [9–11]. Thus, there is an urgent need for global surveillance of antimicrobial resistance as this is important in the management of children with diarrhoea [12].

Despite diarrhoea in children being acknowledged as a serious public health problem in Zambia, there is a paucity of data on infectious diarrhoeal agents, especially bacterial pathogens and their antimicrobial susceptibility patterns due to the few studies that have been conducted in the country [13–15]. Since aetiological agents and drug resistance patterns vary greatly across countries, regions and communities over time, current local knowledge of these patterns is essential to inform treatment, prevention and control programmes. Therefore, the aim of this study was to identify bacterial pathogens in stool samples obtained from children aged 0–59 months admitted with diarrhea to the University Teaching Hospital (UTH) in Lusaka, Zambia.

## Methods

### Setting

The study was conducted at the University Teaching Hospital (UTH), a tertiary referral and teaching hospital in Lusaka with a bed capacity of approximately 2000, and is also the reference centre for all microbiology diagnostic work in Zambia.

### Type of study

This was a cross sectional study. Stool samples were collected from children aged 0–59 months with diarrhoea who attended the UTH from December 2015 to April 2016. Samples were submitted to the microbiology laboratory to determine the presence of bacterial enteropathogens and their antimicrobial susceptibility patterns.

### Isolation and identification of bacterial enteropathogens

**Fig. 1:**
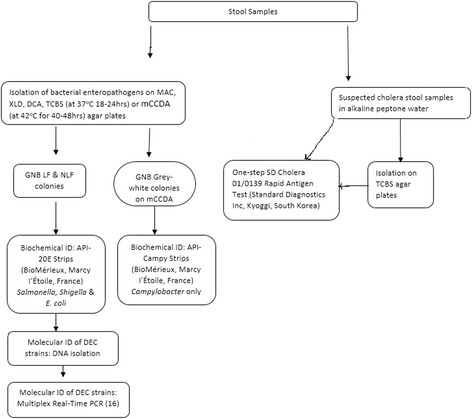
Flow chart showing the isolation and identification of bacterial enteropathogens

Stool samples were inoculated onto MacConkey, Deoxycholate Citrate Agar (DCA) and Xylose Lysine Deoxycholate (XLD) agar plates (Mast Diagnostics Ltd, Merseyside, UK). Samples from suspected cases of cholera were first inoculated into alkaline peptone water and subcultured onto Thiosulphate Citrate Bile Salt (TCBS) (Mast Diagnostics Ltd, Merseyside, UK). All cultures were incubated at 35–37 °C for 12–18 h except for the modified Charcoal-Cefoperazone Deoxycholate Agar (mCCDA) plates (Himedia, Mumbai, India), which were incubated at 42 °C for 72 h in a candle jar sealed with parafilm (Parafilm M, Pechiney Plastic Packaging, Chicago, USA). *C. jejuni* ATCC 33291 strain was used as a positive control. Figure 1 show the approach used for the identification process for the bacterial enteropathogens.

For the identification of diarrhoeagenic *Escherichia coli* (DEC), DNA from the isolates was extracted on the easyMag instrument (bioMérieux, Marcy I’Etoile, France) according to the manufacturer’s instructions using the “on-board lysis” protocol. DNA was eluted in a final volume of 110 μl. All isolates identified as *E. coli* were screened further for virulence genes by multiplex real-time Polymerase Chain Reaction (PCR) targeting specific genes that are associated with six different pathotypes of DEC: enteropathogenic *E. coli* (ETEC), enteroinvasive *E. coli* (EIEC), enteropathogenic *E. coli* (EPEC), enteroaggregative *E. coli* (EAEC), Shiga toxin-producing *E. coli* (STEC and diffusely adherent *E. coli* (DAEC). The minimum criteria for determining diarrhoeagenic *E. coli* were defined as the presence of *stIa*/*stIb* and *lt* for ETEC, presence of *ipaH* for EIEC, presence of *eaeA* for EPEC, presence of *aggR* for EAEC, *stx* 1 and *stx* 2 for STEC and *daaD* for DAEC [16]. The PCR assay was carried out as previously described [16]. *E. coli* DH5α, which lacks all the diarrhoeagenic genes, was used as a negative control. The PCR was performed on an Applied Biosystems 7500 Real-Time PCR cycler (Life Technologies, California, USA) (Fig. 1).

### Antimicrobial susceptibility

This was performed by the Kirby-Bauer disc diffusion method using the CLSI guidelines [17] on Müeller-Hinton agar plates (Mast Diagnostics Ltd, Merseyside, UK) (Table 1). Isolates of *E. coli* were also screened for Extended Spectrum β-Lactamases (ESBL) production by testing them against cefpodoxime (10 μg), ceftazidime (30 μg), and cefotaxime (30 μg) (Mast Diagnostics Ltd, Merseyside, UK) as indicator cephalosporins [18]. Production of ESBLs was confirmed phenotypically by using the combination discs on Müeller-Hinton agar plates (Mast Diagnostics Ltd, Merseyside, UK): cefotaxime/clavulanic acid, cefpodoxime/clavulanic acid and ceftazidime/clavulanic acid (Mast Diagnostics Ltd, Merseyside, UK). After incubation for 18–24 h at 37 °C, the zones of inhibition of the indicator cephalosporin and cephalosporin/clavulanic acid were measured using Vernier callipers and compared. Confirmation of ESBL production was indicated by the zone size of the cephalosporin/clavulanic acid being greater than the indicator cephalosporin (i.e., ≥5 mm). *E. coli* ATCC 25922 and *S. aureus* ATCC 25923 were used as quality control strains for susceptibility testing.

**Table 1:** Antibiotic used in this study and their concentrations

Antibiotic	Abbrev	Conc (μg)	Bacterial pathogen				
			*V. cholerae*	*Salmonella*	*E. coli*	*Shigella*	*Campylobacter*
Amoxillin/clavulanic acid	AMC	20	N	Y	N	Y	N
Ampicilin	AMP	10	Y	Y	Y	Y	Y
Azithromycin	AZM	15	Y	Y	N	Y	N
Cefdoxime	CPD	10	N	N	Y	N	N
Cefotaxime	CTX	30	Y	Y	Y	Y	N
Ceftazidime	CAZ	30	N	N	Y	N	N
Chloramphenicol	CHL	30	Y	Y	Y	Y	Y
Ciprofloxacin	CIP	5	Y	Y	Y	N	Y
Clindamycin	CLI	10	N	N	N	N	Y
Colistin	CST	25	N	Y	N	Y	N
Co-trimoxazole	CMX	25	Y	Y	Y	Y	Y
Doxycycline	DOX	30	N	N	N	N	Y
Erythromycin	ERY	15	Y	Y	N	N	Y
Gentamicin	GEN	10	Y	Y	N	Y	Y
Nalidixic acid	NAL	30	Y	Y	Y	Y	Y
Neomycin	NEO	10	N	Y	N	Y	N
Nitrofurantoin	NIT	30	Y	N	N	N	N
Norfloxacin	NOR	10	Y	Y	N	N	Y
Spectinomycin	SPT	25	N	Y	N	Y	N
Streptomycin	STR	10	N	Y	Y	Y	N
Tetracycline	TET	30	Y	Y	Y	Y	Y

*Abbrev* abbreviations, *Y* yes, *N* no

### Data analysis

Data analysis was performed with GraphPad Prism Software Version 5.0 for Windows (GraphPad Software, San Diego, California, USA) and EpiInfo Version 7.1.5 Software (CDC, Atlanta, USA). Descriptive data analysis was utilized to determine the range of enteropathogens and distribution of study covariates.

## Results

### Isolation and identification of bacterial enteropathogens

Of the 271 children enrolled, 54.6% of them were boys, while the rest were girls (45.4%). Most of the children were less than 24 months (61.3%), followed by those aged between 48 and 59 months (15.5%), 24 to 35 months (12.6%) and 36 to 47 months (10.7%) (Table 2).

**Table 2:** Number of diarrhoeal cases identified, subdivided according to child age and gender

Characteristic	n (%)	Bacterial enteropathogens				
		*V. cholerae*	*Salmonella*	*E. coli*	*Shigella*	*Campylobacter*
		n (%)	n (%)	n (%)	n (%)	n (%)
Gender						
Male	48 (56.5)	23 (27.1)	12 (14.1)	6 (7.1)	6 (7.1)	1 (1.2)
Female	37 (43.5)	11 (12.9)	9 (10.6)	9 (10.6)	6 (7.1)	2 (2.3)
Age group (months)						
< 12	14 (16.5	2 (5.9)	4 (19.0)	6 (40.0)	2 (16.7)	0 (0.0)
12–23	28 (32.9)	13 (38.2)	10 (47.6)	3 (20.0)	1 (8.3)	1 (33.3)
24–35	11 912.9)	5 (14.7)	4 (19.0)	1 (6.7)	1 (8.3)	0 (0.0)
36–47	13 (15.3)	7 (20.6)	1 (4.8)	2 (13.3)	3 (25.0)	0 (0.0)
48–59	19 (22.4)	7 (20.6)	2 (9.5)	3 (20.0)	5 (41.7)	2 (66.7)

Culture results showed that 31.4% of the stool samples analysed were positive for five different bacterial enteropathogens: *Vibrio cholerae* (40.8%), *Salmonella* species (25.5%), DEC (18%), *Shigella* species (14.4%) and *Campylobacte*r species (3.5%) (Table 3). All the *V. cholerae* isolates detected were of the 01 subtype and Ogawa serotype. Amongst the *Salmonella* species, 52.4%) were *S*. Typhi, 19.1% were *S.* Paratyphi B and 28.6% were Non-Typhoidal *Salmonella* (NTS). Of the DEC detected, the most frequent was ETEC (40%), followed by EIEC (26.7%), EAEC (20%), and EPEC (13.3%). No STEC or DEAC strains were detected. Of the 12 *Shigella* isolates, 50% were *Shigella flexneri*, 33.3% were *Shigella dysenteriae* and 16.7% were *Shigella boydii. Campylobacter jejuni* comprised only 3.5% of the total number of isolates (Table 3).

**Table 3:** Distribution of bacterial enteropathogens isolated stool specimens

Bacterial enteropathogen	n (%)
*Vibrio cholerae* 01 (Ogawa)	34 (40.8)
*Salmonella* species	21 (25.2)
*Salmonella* Typhi	11 (52.4)
*Salmonella* Paratyphi B	4 (19.1)
Other *Salmonellae*	6 (28.6)
*Escherichia coli* (DEC)	15 (18.0)
ETEC	6 (40.0)
EIEC	4 (26.7)
EAEC	3 (20.0)
EPEC	2 (13.3)
*Shigella* species	12 (14.4)
*Shigella flexneri*	6 (50.0)
*Shigella dysenteriae*	4 (33.3)
*Shigella boydii*	2 (16.7)
*Campylobacter jejuni*	3 (3.5)

*DEC* diarrhoeagenic *E. coli*, *ETEC* enterotoxigenic *E. coli*, *EIEC* enteroinvasive *E. coli*, *EAEC* enteroaggregative *E. coli*, enteropathogenic *E. coli*, *n* number

*Vibrio cholerae* and *Salmonella* species were most commonly recovered from children aged between 12 and 23 months. The most prevalent pathogen, *V. cholerae*, was detected mainly in children older than 12 months. DEC mainly affected children less than 12 months, while *Shigella* mainly affected children older than 36 months.

### Antimicrobial susceptibility patterns

All *V. cholerae* isolates exhibited 100% resistance to co-trimoxazole, nalidixic acid and nurafurantoin. The isolates showed low level of resistance to erythromycin (32.4%), ciprofloxacin (26.5%), norfloxacin (20.5%) and chloramphenicol (8.8%). All the isolates were 100% susceptible azithromycin, ampicillin, cefotaxime and gentamicin, while the strains were 94.1% sensitive and 5.9% intermediate to tetracycline.

We also attempted to find antimicrobial resistance patterns of the bacterial enteropathogens. Multidrug resistance (MDR) was defined as resistance to three or more drugs. The majority of the *V. cholera* (97%) were MDR with 9 different patterns. The most common pattern was ciprofloxacin-cotrimoxazole-erythromycin-nalidixic acid (11.8%), followed by ciprofloxacin-cotrimoxazole-nalidixic acid-norfloxacin (8.8%) and cotrimoxazole-erythromycin-nalidixic acid (5.9%) (Table 4).

**Table 4:** Antimicrobial resistance pattern of *V. cholerae*

Antimicrobial resistance pattern	*V. cholerae* (34) (n,%)
CHL-CMX-ERY-NAL	1(2.9)
CHL-CMX -NAL-NOR	1(2.9)
CIP-CHL-CMX-NAL	1(2.9)
CIP-CMX-ERY-NAL	4(11.8)
CIP-CMX-NAL-NOR	3(8.8)
CMX-ERY-NAL-NOR	1(2.9)
CIP- CMX-NAL	1(2.9)
CMX-ERY-NAL	2(5.9)
CMX-NAL-NOR	1(2.9)
Total MDR	15(97)

*S.* Typhi species displayed 100% resistance to ampicillin, co-trimoxazole and streptomycin, 72.7% to chloramphenicol, 18.2% to azithromycin and 9.1% to ciprofloxacin. The isolates isolates had four different MDR patterns, the commonest being ampicillin-chloramphenicol-cotrimoxazole-streptomycin (45/5%), followed by ampicillin-cotrimoxazole-streptomycin (36.4%), ampicillin-azithromycin-chloramphenicol-cotrimoxazole-streptomycin (18.2%) and ampicillin-chloramphenicol-ciprofloxacin-streptomycin (9.1%). However, these isolates were all susceptible to nalidixic acid, amoxycylin-clavulanic acid, tetracycline, spectinomycin, gentamicin, cefotaxime, neomycin and colistin. *S.* Paratyphi B isolates were 100% resistant to ampicillin, co-trimoxazole and streptomycin, chloramphenicol and 75% resistant to spectinomycin. Its MDR patterns were ampicillin-chloramphenicol-cotrimoxazole-spectinomycin-streptomycin (75%) and ampicillin-chloramphenicol-cotrimoxazole-streptomycin (25%). This group of *Salmonella* isolates were susceptible to azithromycin, ciprofloxacin, nalidixic acid, amoxycylin-clavulanic acid, gentamicin, cefotaxime, neomycin and colistin. The NTS isolates showed the following resistance patterns: 100% to co-trimoxazole, 88.3% to ampicillin, 66.7% to streptomycin, 50% to chloramphenicol, 33.3% to spectinomycin, and 16.7% to both colistin and tetracycline. The group had six different patterns each exhibiting 16.7% (Table 5). These isolates were also susceptible to the following antibiotics: to azithromycin, ciprofloxacin, nalidixic acid, amoxycylin-clavulanic acid, gentamicin, cefotaxime and neomycin.

**Table 5:** MDR patterns for Salmonella species [21]

Antimicrobial resistance pattern	S. Typhi (11) (n,%)	S. Paratyphi B (4) (n,%)	NTS (6) (n,%)
AMP-AZM-CHL-CMX-STR	2(18.2)		
AMP-CHL-CIP-CMX-STR	1(9.1)		
AMP-CHL-CMX-SPN-STR		3(75)	1(16.7)
AMP-CHL-CMX-STR-TET			1(16.7)
AMP-CHL-CMX-STR	5(45.5)	1(25)	
AMP-COL-CMX-STR			1(16.7)
CMX-SPN-STR			1(16.7)
AMP- CMX-STR	3(36.4)		

DEC generally exhibited high rates of resistance to most antibiotics tested. ETEC strains were more resistant to co-trimoxazole (100%), followed by ampicillin and cefotaxime (66.7%), ceftazidime (66.7%), cefpodoxime (66.7%), tetracycline (both 50%), streptomycin (33.7), chloramphenicol and nalidixic acid (both 16.4%). The four types of DEC isolated showed 100% resistance to co-trimoxazole and each type was 50% resistant to tetracycline. The most resistant strains belonged to the EPEC group. The stains displayed the following MDR patterns: ETEC, six; EIEC, four; EAEC, three; and EPEC, two (Table 6).

**Table 6:** *E.coli* strains

Antimicrobial resistance pattern	ETEC (6) (n,%)	EIEC (4) (n,%)	EAEC (3) (n,%)	EPEC (2) (n,%)
AMP-CAZ-CMX-CPD-CTX-NAL-STR-TET	1(16.7)			
AMP-CAZ-CMX-CPD-CTX-NAL-TET		1(25)		
AMP-CAZ-CMX-CPD-CTX-STREP	1(16.7)			
AMP-CAZ-CMX-CPD-CTX-TET	1(16.7)		1(33.3)	1(50)
AMP-CMX-NAL-STR-TET		1(25)		
AMP-CAZ-CMX-CPD-CTX		1(25)		1(50)
CAZ-CMX-CPD-CTX	1(16.7)			
AMP-CHL-CMX	1(16.7)		1(33.3)	
AMP-CMX-TET	1(16.7)			
AMP-CMX-STR			1(33.3)	
AMP-CMX-NAL		1(25)		

After analysing all the 15 isolates of DEC for ESBL production, 66.7% (10/15) were found to be resistant to cefotaxime, ceftazidime and cefpodoxime, suggesting that they were potential producers of ESBL. Further analysis of the isolates with a confirmatory test (combination discs: cefotaxime-clavulanic acid, ceftazidime clavulanic acid and cefpodoxime clavulanic acid) showed that all of them were ESBL-producers.

Among the *Shigella* species, *S. flexneri* was resistant to ampicillin and co-trimoxazole (both 100%), followed by chloramphenicol and streptomycin (both 83.8%), amoxicillin-clavulanic acid, and tetracycline (both 16.5%). *S. dysenteriae* displayed 100% resistance to both ampicillin and co-trimoxazole (75%), to chloramphenicol (25%) and 25% to tetracycline. *S. bodii* was 100% resistant to ampicillin, co-trimoxazole and chloramphenicol. Their MDR patterns were as follows: *flexneri*, 4, the commonest being ampicillin-chloramphenicol-cotrimoxazole-streptomycin (50%); *S. dysenteriae*, 2, with ampicillin-chloramphenicol-cotrimoxazole-streptomycin being the commonest (75%); and both *S. boydii* displayed only two different patterns, ampicillin-chloramphenicol-cotrimoxazole (100%) (Table 7).

**Table 7:** Antimicrobial resistance pattern of Salmonella species

Antimicrobial resistance pattern	*S. flexneri* (6)	*S. dysenteriae* (4)	*S. boydii* (2)
AMP-AMC-CHL-CMX-STR	1(16.7)		
AMP-CHL-CMX-STR-TET	1(16.7)	1(25)	2(100)
AMP-CHL-CMX-STR	3(50)	1(75)	
AMP-CHL-CMX	1(16.7)		

The three *Campylobacter jejuni* species isolated were only resistant to co-trimoxazole (100%), ampicillin (33.3%) and tetracycline (33.3%).

## Discussion

Five different bacterial enteropathogens were isolated from some of the stool specimens, and these included *V. cholerae* 01 Ogawa serotype, *Salmonella* species, *Shigella* species, DEC and *Campylobacter* species. *V. cholerae* was the most predominant pathogen due to a cholera outbreak during the study period. Our results were in agreement with previous studies done in Lusaka [19, 20]. However, none of these studies focused on cholera in children.

*Salmonella* species was the second most prevalent genera (25.5%); and the majority of the species were S. Typhi followed by NTS and *S*. Paratyphi B, which agrees with other studies conducted in India and Bangladesh [21–23].

Among the DEC isolates, four strains were identified: ETEC, EIEC, EAEC and EPEC, with EPEC being the most predominant species. Four strains of DEC (ETEC, EIAC, EAEC and EPEC) were the third most common group of enteropathogens, accounting for 18.0% of all isolates. The predominance of ETEC strain, among the DEC, in agreement with previous studies carried out in Bangladesh, Tunisia and Kenya [24–26]. ETEC was associated with one third of diarrhoea cases identified in children in the recent GEMS Study [4].

DEC isolates were mainly recovered from children below the age of 24 months, which corroborates with the findings of a Nigerian study that also indicated that most of the DEC isolates were mostly recovered from this age group [27]. In this study, EAEC was detected from only 3 specimens, and EIEC and EPEC strains were also detected but in low numbers.

*Shigella* species ranked third, with the most predominant species being *S. flexneri*, and affected mainly children above 36 months of age. This finding was in conformity with previous studies that have implicated *S. flexneri* as the dominant species [28, 29]. However, other studies have indicated that *S. dysenteriae* and *S. boydii* to be more frequently isolated species [28–30].

Only three *Campylobacter* species were recovered from the children, which might probably be due to the fact that *Campylobacter* is as a fastidious organism which requires special conditions for growth [31]. However, in a community-based study on the pathogen-specific burden of diarrhoea in low-income countries, that involved eight study sites in South America, Africa and Asia, *Campylobacter* exhibited the highest attributable burden of diarrhoea amongst infants aged between 0 and 11 months [31].

In this study, all the organisms isolated exhibited high level drug resistance, including resistance to multiple drugs. *V. cholerae* isolates were totally resistant to cotrimoxazole and nalidixic acid, partially resistant to erythromycin, ciprofloxacin and norfloxacin. A previous study in Zambia, during the 1990–1991 major cholera outbreak, showed resistant to cotrimoxazole (97%), tetracycline (95%), chloramphenicol (98%) and doxycycline (70%) [19]. A similar study in Mozambique, found that the *V. cholerae* O1 Ogawa isolates were resistant to cotrimoxazole (100%), ampicillin (100%), nalidixic acid [9], chloramphenicol (97%), nitrofurantoin (95%), tetracycline (82%), azithromycin (56%) but sensitive to ciprofloxacin (100%) (101).

For the *Salmonella* species, both *S*. Typhi and *S.* Paratyphi B were totally resistant to ampicillin, cotrimoxazole and streptomycin. In addition to the three drugs, *S.* Typhi and *S.* Paratyphi B were also significantly resistant to chloramphenicol and spectinomycin, respectively. These findings are consistent with other studies in which it was noted that there was an increase in the number of *Salmonella* isolates being resistant to ampicillin, chloramphenicol and cotrimoxazole [32].

A study on the Malawi-Mozambique border reported findings similar to those in this study in which 100% of *S.* Typhi were resistant to ampicillin, chloramphenicol and cotrimoxazole [33]. Another study carried out in Uganda showed that 76% of *S.* Typhi isolates were resistant to ampicillin, streptomycin, sulfisoxazole, tetracycline, and cotrimoxazole, but were susceptible to chloramphenicol. This was in conformity with findings from this study which also found that all the *S.* Typhi isolates were susceptible to ceftaxime, gentamicin, and spectinomycin [34].

This study also demonstrated the occurrence of MDR strains of *S.* Typhi, *S.* Paratyphi B and the NTS which were resistant to all traditional first line drugs tested: ampicillin, chloramphenicol and cotrimoxazole. The commonest resistance pattern observed in this study was ampicillin-chloramphenicol-cotrimoxazole-streptomycin for *S.* Typhi, ampicillin-chloramphenicol-spectinomycin-cotrimoxazole- streptomycin for *S.* Paratyphi B, while NTS has 6 different patterns, which also included the ampicillin-chloramphenicol-spectinomycin-cotrimoxazole- streptomycin pattern. A similar study by Demczuk and colleagues [35], revealed 26 resistance patterns with the commonest patterns being nalidixic acid-resistant (NAR) and ampicillin-chloramphenicol-nalidixic acid-streptomycin-sulfisoxazole-cotrimoxazole in *S*. Typhi compared to this to this study. This study indicated that most of the *Salmonella* isolates were resistant to two or more antibiotics and there was variability in the resistant patterns of NTS. The MDR detection rates *S*. Typhi, *S.* Paratyphi B, and the NTS were all 100%. Similar findings of MDR strains (100%) were reported in Turkey [36].

All the DEC isolates were highly resistant to cotrimoxazole, ampicillin, cefotaxime, ceftazidime, cefpodoxime, nalidixic acid, and tetracycline. Generally, the isolates showed moderate to high resistance to most drugs, and low resistance to streptomycin and chloramphenicol. Our data suggests the presence of MDR in all the DEC isolates (100%) recovered in this study. This high prevalence of MDR strains observed in this and other studies is worrisome in that it limits treatment options for the patients. Another important observation in this study with the DEC isolates was that they were all resistant to third generation cephalosporins used. Interesting, all the isolates proved to be ESBL-producers. The detection of ESBL-producing DEC isolates warrants attention in the context of increasing resistance amongst the enteropathogens. The high prevalence of ESBL in this study may suggest over prescription of third generation cephalosporins in or inappropriate use of antibiotics in Lusaka.

In this study all the *Shigella* species were mainly resistant to ampicillin, cotrimoxazole, chloramphenicol and streptomycin, although *S. boydii* was sensitive to streptomycin. The common resistance pattern was ampicillin-chloramphenicol-cotrimoxazole-streptomycin. These data suggest the presence of MDR in all the *Shigella* isolates (100%) recovered. This MDR pattern was in consonance with our study.

The two *Campylobacter* species isolated in this study were resistant to co-trimoxazole and moderately resistant to both ampicillin and tetracycline. In a Zimbabwean study, 50% of the isolates from humans and 82% from chickens were resistant to co-trimoxazole, while for ampicillin and tetracycline the levels were similar as those in this study. However, MDR resistant isolates were consistently susceptible to erythromycin, chloramphenicol and gentamicin [37].

## Conclusion

This study isolated and identified five enteric bacteria from stools obtained from children with diarrhea and the majority of these organisms exhibited drug resistance to ampicillin and co-trimoxazole. This presents very limited treatment options for patients, necessitating a review of empirical treatment practices at the UTH to avoid further complications in affected patients. The study however, recommend good hygienic practices in different communities through public health educational programme in order to avoid cases diarrhoea infections among children.

## Abbreviations

CDC: Centres for Disease Control and Prevention
DEC: Diarrhoeagenic *Escherichia coli*
DNA: Deoxy Ribonucleic Acid
ERES: Excellence in Research Ethics and Science
ESBL: Extended Spectrum of Beta Lactamase
IRB: Institutional Review Board
MDR: Multi Drug Resistance
MUHAS: Muhimbili University of Health and Allied Sciences
NTS: Non Typoidal Salmonella
PCR: Polymerase Chain Reaction
TFELTP: Tanzanian Field Epidemiology and Laboratory Management Training Programme
UTH: University Teaching Hospital
